# Impact of Climate Change on the Invasion of *Mikania micrantha* Kunth in China: Predicting Future Distribution Using MaxEnt Modeling

**DOI:** 10.3390/plants14233694

**Published:** 2025-12-04

**Authors:** Chunping Xie, Zhiquan Chen, Mianting Yu, Chi Yung Jim

**Affiliations:** 1 Tropical Biodiversity and Bioresource Utilization Laboratory, Qiongtai Normal University, Haikou 571127, China; xcp@mail.qtnu.edu.cn (C.X.); ymtqtnu@163.com (M.Y.); 2 Haikou Experiment Station, Chinese Academy of Tropical Agricultural Sciences, Haikou 571101, China; 3 Department of Social Sciences and Policy Studies, Education University of Hong Kong, Tai Po, Hong Kong, China

**Keywords:** *Mikania micrantha*, climate change, invasive alien species, MaxEnt modeling, species distribution, spatial containment

## Abstract

Invasive alien species pose escalating threats to global biodiversity and ecosystems, which may be exacerbated by climate change, potentially leading to range expansions and intensified impacts. In China, *Mikania micrantha* Kunth, a fast-growing tropical vine listed among the world’s 100 worst invasive species, has proliferated since its introduction in the mid-20th century, causing severe ecological damage through the smothering of vegetation, suppression of allelopathy, and economic losses in agriculture and forestry. This study aimed to predict its current and future distributions to guide management. Using 205 stringently filtered occurrence records from databases, surveys, and literature, combined with bioclimatic variables from WorldClim and MaxEnt modeling—optimized via ENMeval and evaluated by AUC (>0.97)—projected habitats under current (1970–2000) conditions and future SSP1-2.6, SSP2-4.5, and SSP3-7.0 scenarios for the 2050s and 2070s via the BCC-CSM2-HR model. Temperature factors dominated predictions, with current excellent suitability (3.6 × 10^4^ km^2^) concentrated in Hainan and southern Guangdong, expanding to good and moderate zones in Guangxi, Fujian, and Yunnan. Future averages showed expansions in excellent (21.3%), good (10.0%), and moderate (14.0%) habitats, with some northward shifts into Jiangxi and Hunan under higher emissions. In situ augmentation of habitat suitability and spatial containment overshadows the northward range expansion. The high-emission scenario is projected to lead to temperature overshoots, which will dampen habitat suitability. The findings underscore *M. micrantha*’s resilience to warming, necessitating integrated strategies such as guarding critical biodiversity sites, early detection, biocontrol, and habitat restoration to mitigate risks in both core and emerging zones.

## 1. Introduction

**Figure 1:**
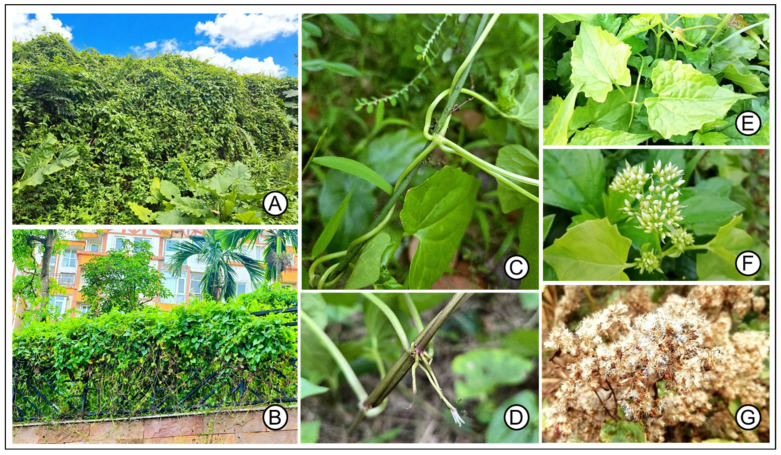
Current invasion status and morphological characteristics of *Mikania micrantha*. Recent field surveys show that *M. micrantha* has become rampant in both rural (**A**) and urban (**B**) areas of tropical China. The tropical perennial herbaceous climber has twining stems (**C**) and well-developed aerial and adventitious roots (**D**). It exhibits vigorous and rampant growth, along with strong straggling behavior that can rapidly climb and cover many host plants, eventually causing their death. Panels (**E**–**G**) show the leaves, flowers, and fruits of *M. micrantha*, respectively. Its prolific seeds are small and light (about 2 mm long) and bear a parachute-like pappus at the base, facilitating long-distance dispersal by wind, water, animals, insects, and human activities [10].

Invasive alien species (IAS) represent one of the most pressing threats to global biodiversity, ecosystem stability, and economic productivity, particularly in the context of accelerating climate change [1,2]. These species exploit disturbed environmental conditions to expand their ranges, often outcompeting native flora and fauna, disrupting nutrient cycles, and imposing substantial productivity losses and financial burdens on agriculture and land management [3,4]. Climate change exacerbates the impacts by modifying temperature regimes, precipitation patterns, and extreme weather events, thereby creating novel habitats that favor IAS proliferation and enabling shifts in their distributional limits toward higher latitudes and elevations [5,6]. Endowed with biodiversity hotspots and diverse climatic zones, China’s interplay between IAS invasions and climate change poses an escalating risk to native ecosystems, agricultural systems, and socioeconomic sectors [7]. Among these invaders, *Mikania micrantha* Kunth (commonly known as mile-a-minute weed) stands out as a particularly pernicious species [8], warranting urgent attention due to its rapid spread and multifaceted impacts (Figure 1) [9].

Native to tropical and subtropical Central and South America [11], *M. micrantha* (Asteraceae) is a fast-growing perennial vine that has established itself as one of the world’s 100 worst invasive alien species, according to the International Union for Conservation of Nature (IUCN) [9,12]. Introduced inadvertently through trade and horticulture [8,13], it has proliferated across Asia, Africa, and the Pacific, where it rapidly climbs trees and shrubs to form dense mats that rest on canopy tops. The hosts will soon be smothered and killed by reduced light penetration, physical obstruction of growth, and altered soil chemistry [14,15]. Its rapid growth is facilitated by photosynthetic stems and adventitious roots [16]. The plant’s allelopathic properties release secondary metabolites that inhibit the growth of nearby species by suppressing germination and seedling growth [9,12,17], thereby amplifying its competitive advantage. Invaded areas will experience significant declines in native plant biomass and diversity, as well as disruptions to the ecological balance [12]. Economically, *M. micrantha* causes severe damage to agricultural crops, forestry plantations, and orchards by entwining and suppressing host plants, resulting in yield losses estimated at millions of dollars annually [8,18]. Its impacts extend to ecosystem services [9], as infested areas experience degraded soil structure and disrupted nutrient cycling [15].

The aggressive weed has invaded large areas in Asia, including Southeast Asia and the Pacific region. In China, *M. micrantha* has been spreading and invading widely since the 1980s, covering vast expanses of Guangdong, Yunnan, and other subtropical areas, and transforming forests, grasslands, and farmlands into monoculture-like stands [19]. This rapid and widespread invasion has led to profound ecological consequences [18], including the displacement of endemic species, heightened vulnerability to secondary invasions, and increased wildfire risks due to the accumulation of dry biomass. Socioeconomically, the plant poses a threat to key agro-industries, including tea, rubber, and fruit production [18]. Control measures, ranging from manual removal to chemical herbicides, incur high costs and provide only temporary relief [20,21]. This limitation stems from the plant’s prodigious reproductive success, which is attributed to its prolific seed production (up to 40,000 seeds per plant annually), extensive seed dispersal, high germination rate, and effective vegetative propagation [19,22]. The urgency for effective management is underscored by the species’ resistance and resilience to current control measures, as well as its potential to exacerbate food security challenges in a nation grappling with land degradation and population pressures [23]. Without proactive and effective intervention, *M. micrantha* could perpetuate a cycle of biodiversity loss and economic strain, emphasizing the need for integrated strategies that incorporate early detection, rapid response, and long-term monitoring.

Climate change introduces an additional layer of complexity to *M. micrantha*’s invasion dynamics in China [24,25]. Projected increases in temperature and alterations in rainfall patterns are likely to expand suitable habitats northward, potentially invading temperate zones previously deemed unsuitable [26]. Such range extensions could exacerbate interactions with native vegetation and species, amplify allelopathic effects under stress conditions, and accentuate the plant’s reproductive success, thereby accelerating invasion rates and spreads [27]. Despite growing recognition of these risks, empirical predictions of future distributions remain limited, hindering targeted policy formulation and resource allocation. Species distribution models (SDMs), such as the Maximum Entropy (MaxEnt) algorithm [28,29,30,31], offer a robust framework for addressing this gap by integrating occurrence data with bioclimatic variables to forecast habitat suitability under various climate scenarios. MaxEnt’s efficacy in handling presence-only data and accounting for multicollinearity is particularly suitable for invasive species modeling [32], where comprehensive absence records are often unavailable. MaxEnt has achieved notable success in predicting invasive alien species [33,34], providing a scientific foundation for their prevention and management.

Despite the urgent ecological and economic threats posed by *M. micrantha* in China, comprehensive assessments of how changing climates may alter its future distribution remain limited. To date, only one regional MaxEnt prediction exists, which is restricted to Yunnan Province and has limited occurrence records [35]. A national-scale, high-resolution projection that can inform large-scale, prioritized management of this invasive species across its entire invaded range in China is still lacking, hindering the development of effective nationwide prevention and control strategies. The interaction between climate change and invasive species dynamics is complex and context-dependent, requiring spatially explicit, quantitative approaches to inform management priorities. This study addresses this knowledge gap by: (1) determining the current potential distribution of *M. micrantha* across China based on comprehensive data on occurrence and bioclimatic variables; (2) projecting potential future distributions under multiple climate change scenarios; and (3) identifying regions of heightened invasion risk that warrant prioritized monitoring and preventive management. By integrating species occurrence data, environmental characterization, and climate projection models, this research aims to provide evidence-based guidance for stakeholders in agriculture, forestry, and conservation management. The findings can enable more effective strategies to mitigate the escalating *M. micrantha* invasion in the face of global environmental change.

## 2. Results

### 2.1. Model Accuracy

**Figure 2:**
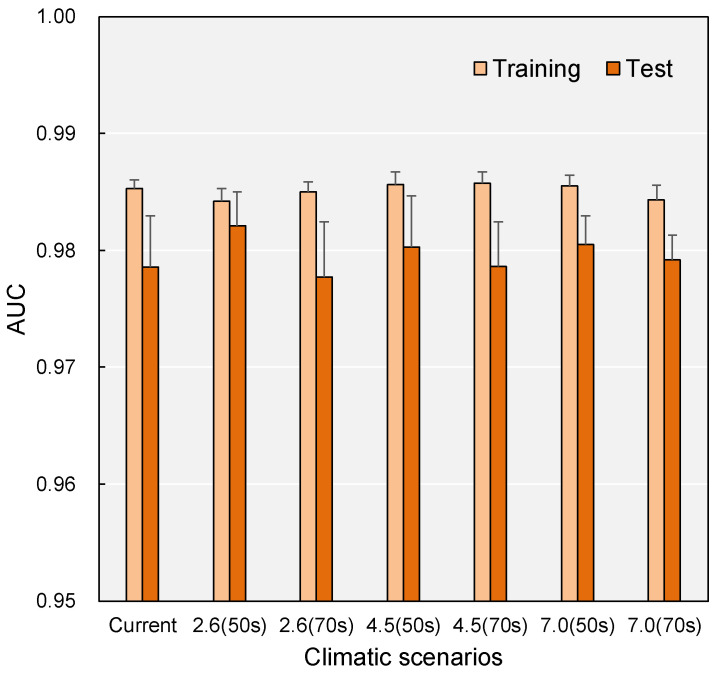
AUC values of habitat distribution under the current climate and three future climate scenarios (SSP1-2.6, SSP2-4.5, and SSP3-7.0), each with two timeframes (2050s and 2070s). Error bars indicate standard deviations.

The AUC values for the habitat distribution of *M. micrantha* across different climate change scenarios and periods indicate strong model performance (AUC > 0.9) (Figure 2), suggesting reliable predictions of species distribution. In the current climate, the training AUC value is 0.985 ± 0.001, and the test AUC is 0.979 ± 0.004, both indicating excellent performance. Under future climate scenarios, the model’s performance remains strong, with training AUC values ranging from 0.984 to 0.986 (±0.001) and test AUC values from 0.978 to 0.982 (±0.002–0.005) across all SSP scenarios and time periods. These high AUC values indicate that the MaxEnt model provides consistent and reliable predictions of *M. micrantha* habitat distribution under current and future climate change conditions.

### 2.2. Key Bioclimatic Drivers of the Current Distribution Pattern

**Figure 3:**
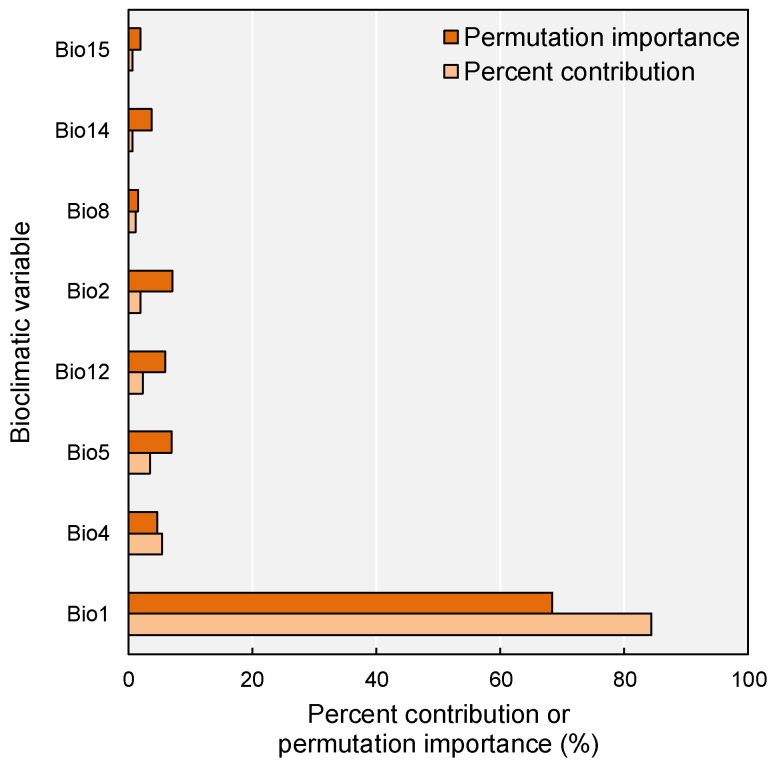
Percentage contribution and permutation importance of the most important bioclimatic variables that contribute more than 1%. Refer to Table A1 for the meaning of the bioclimatic variables.

**Figure 4:**
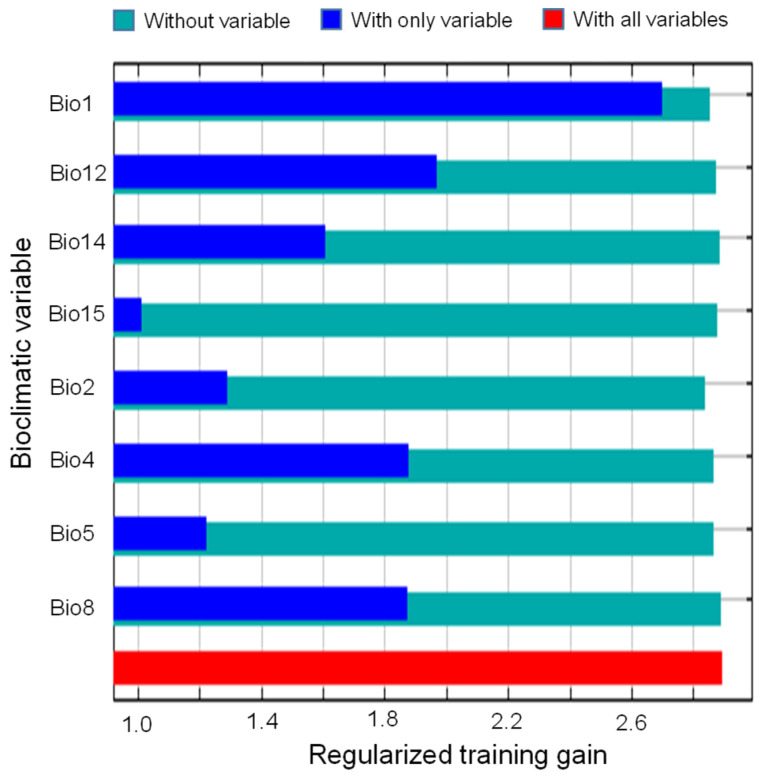
The jackknife of the regularized training gain for the eight most important bioclimatic variables. Refer to Table A1 for the meaning of the bioclimatic variables.

Integrating three complementary metrics (percent contribution, permutation importance, and jackknife regularized training gain), this study identified eight important bioclimatic determinants governing the potential distribution of *M. micrantha* in China (Figure 3; Table A1). The annual mean temperature (Bio1) emerged as the overwhelmingly dominant predictor, accounting for 84.3% of the model’s contribution and 68.3% of the permutation importance, underscoring its preeminent role in delimiting the species’ suitable habitat. Temperature seasonality (Bio4) ranked second in both metrics (5.4% and 4.7%, respectively), highlighting the significance of thermal stability as a secondary constraint. The mean temperature of the warmest month (Bio5) contributed 3.5% with a permutation importance of 6.9%. In comparison, annual precipitation (Bio12) showed inverse patterns (2.3% contribution vs. 5.9% importance), suggesting its critical role in model prediction accuracy despite moderate contribution. Mean diurnal temperature range (Bio2) exhibited notably higher permutation importance (7.1%) relative to its contribution (2.0%), indicating its predictive value when other variables are constrained. Jackknife resampling (Figure 4) further revealed that Bio1, Bio12, and Bio14 produced the largest independent information gain, corroborating the pre-eminent role of thermal conditions and moisture thresholds in delimiting *M. micrantha*’s potential range. Together, the top five variables (Bio1, Bio4, Bio5, Bio12, and Bio2) cumulatively accounted for 97.5% of the model’s explanatory power, with temperature-related variables (Bio1, Bio4, Bio5, Bio2, and Bio8) dominating the climatic niche of *M. micrantha*.

**Figure 5:**
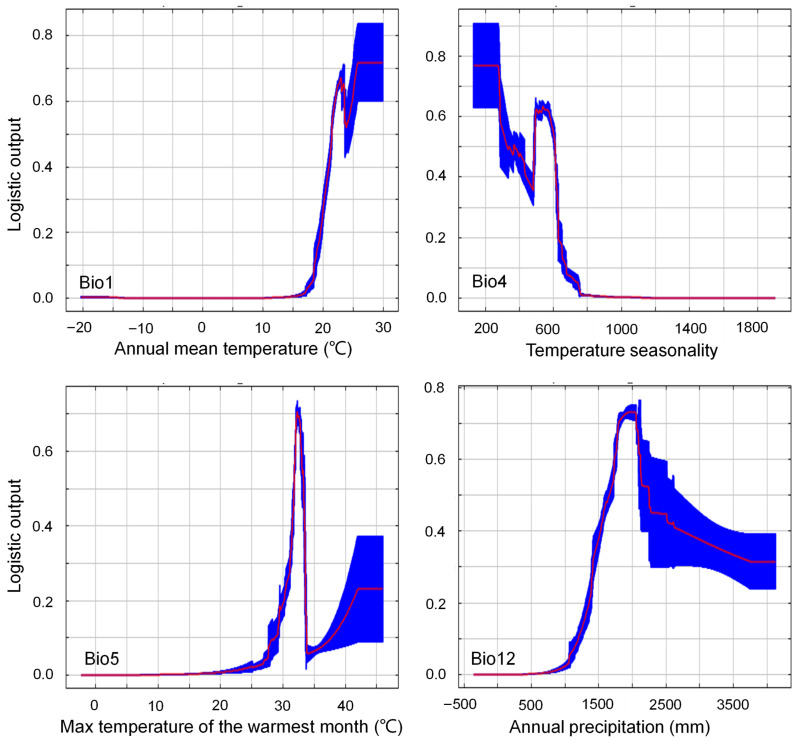
Response curves of the top four bioclimatic predictor variables used in the MaxEnt model for *Mikania micrantha*. The curves show the mean response of the 10 replicate MaxEnt runs (red line) and the mean +/− one standard deviation (blue belt). Temperature seasonality is expressed in the standard deviation unit. Refer to Table A1 for the meaning of the bioclimatic variables.

The response curves illustrate the relationship between key bioclimatic variables and habitat suitability for *M. micrantha*, with red lines representing mean responses across 10 replicate runs and blue belts indicating ±1 standard deviation (Figure 5). A distribution probability > 0.50 is considered suitable for species establishment.

The most influential variable, annual mean temperature (Bio1), exhibited a pronounced sigmoid response curve with a sharp habitat suitability threshold near 20 °C. Suitability remained at a minimal level (<0.10) below 15 °C, increased dramatically to 15–25 °C, and ultimately plateaued at high suitability (>0.80) above 25 °C. The narrow blue margin suggested high prediction confidence, confirming *M. micrantha*’s strong thermophilic characteristics.

Temperature seasonality (Bio4) displayed a contrasting negative relationship, with optimal conditions occurring at low seasonality values (150–400 units, logistic output ~0.85) before declining sharply to near-zero suitability beyond 750 units, indicating the species’ high sensitivity to temperature instability in the annual cycle and strong preference for thermally stable environments.

The maximum temperature of the warmest month (Bio5) showed a unimodal response with a distinct optimal range around 32 °C (logistic output ~0.75), followed by a precipitous decline above 36 °C, indicating a well-defined upper thermal limit beyond which extreme heat imposes physiological constraints. However, the broader blue margin at higher temperatures reflects greater uncertainty in prediction in this extrapolated range.

Annual precipitation (Bio12) exhibited a positive sigmoidal response reaching threshold conditions (>0.50) at approximately 1000–1200 mm and maintaining high suitability (0.70–0.75) across a broad range of 1500–4000 mm. The wide blue margin at higher values indicates increased variability, possibly reflecting regional moisture interactions.

Overall, these response curves reveal that *M. micrantha* is optimally adapted to warm (>20 °C), thermally stable (Bio4: 150–400 units), moderately humid (>1200 mm) environments with specific upper thermal limits (~35 °C). The predictions underscore its tropical origin and limited tolerance to seasonal temperature fluctuations, which corroborates its current invasive distribution patterns in China’s southern subtropical zones, where these climatic conditions converge.

### 2.3. Current Potential Distribution and Habitat Suitability

**Figure 6:**
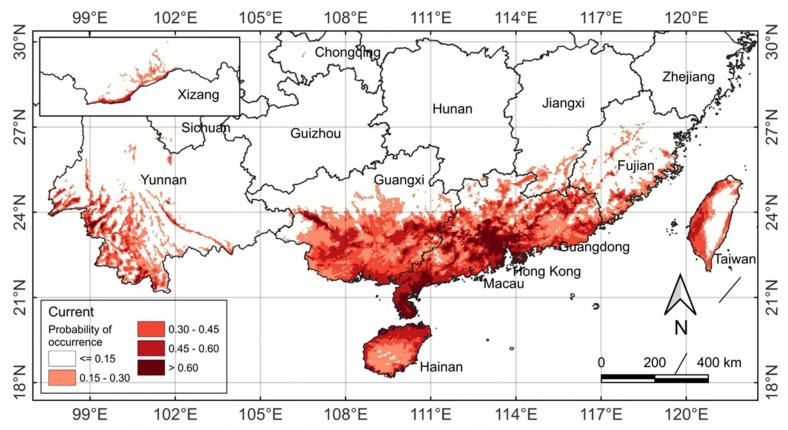
Potential suitability areas of *Mikania micrantha* in China under current climate (1970–2000). Colors represent habitat suitability expressed as logistic probability of occurrence (0–1); areas are classified into five categories: unsuitable (<0.15), low (0.15–0.30), moderate (0.30–0.45), good (0.45–0.60), and excellent (>0.60).

Under the current climate (1970–2000), *M. micrantha* is confined to southern China between approximately 18–32° N and 98–122° E, with dense, highly connected populations in Guangdong, Guangxi, and Hainan provinces and markedly sparser, more fragmented occurrences in Yunnan, Fujian, and Taiwan (Figure 6). Guangdong exhibits the most extensive and continuous invasion, spanning the Pearl River Delta, coastal hubs (Shenzhen, Zhuhai, and Shantou), and inland cities (Meizhou and Shaoguan). Guangxi hosts the second-densest population across its southern plains (Nanning, Beihai, Fangchenggang), thinning northward. On Hainan Island, the species inhabits nearly all lowland areas from Sanya to Haikou, sparing only the central highlands. In contrast, Yunnan is restricted to linear valley hotspots in the southwest (Dehong, Xishuangbanna, Lincang), Fujian and Taiwan to narrow coastal belts (Xiamen, Zhangzhou, Fuzhou, and western Taiwan), and sporadic, non-persistent records occur further north in Jiangxi, Hunan, Guizhou, Chongqing, and Sichuan, largely resulting from human introduction rather than natural spread.

MaxEnt projections identify 3.6 × 10^4^ km^2^ of excellent suitability (>0.60), forming two highly connected core areas that perfectly match the densest observed invasions: (i) nearly the entire Hainan Island plus the Leizhou Peninsula, and (ii) the Pearl River Delta with westward extensions along southern Guangdong (Figure 6; Table 1). Good (8.3 × 10^4^ km^2^) and moderate (10.1 × 10^4^ km^2^) zones surround these cores, dominating the lowland areas of Guangxi and coastal Guangdong, while forming narrow coastal or valley corridors in Fujian, Taiwan, and southwest Yunnan. Low-suitability areas (15.9 × 10^4^ km^2^) occupy the inland and northward fringes, as well as isolated northern outliers, whereas 924.5 × 10^4^ km^2^ of China remain unsuitable, mainly in central, northern, and high-elevation regions.

Overall, the modeled suitability mosaic closely mirrors the observed invasion intensity: excellent and good habitats are almost fully occupied in Guangdong, Guangxi, and Hainan, whereas establishment remains rare or absent in low-suitability northern provinces despite occasional anthropogenic introductions. This tight correspondence confirms that the current Chinese distribution of *M. micrantha* is primarily climate-limited and that the species has already saturated most of its climatically excellent habitats in tropical and south-subtropical China.

### 2.4. Future Habitat Suitability Pattern

**Figure 7:**
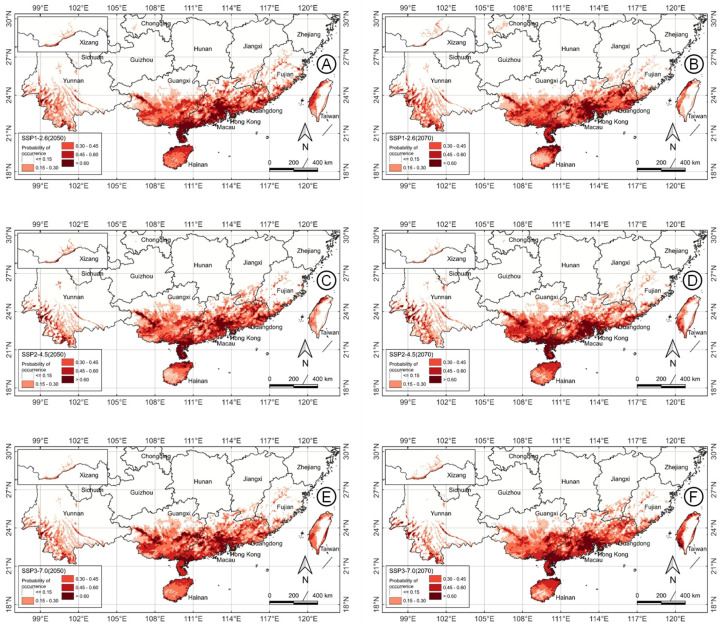
MaxEnt modeling of potential suitability areas for *Mikania micrantha* based on two future climate-change scenarios in the 2050s (**A**,**C**,**E**) and 2070s (**B**,**D**,**F**) (columns) at SSP1-2.6 (**A**,**B**), SSP2-4.5 (**C**,**D**) and SSP3-7.0 (**E**,**F**) (rows). Habitat suitability is divided into five categories based on the calculated habitat suitability index.

MaxEnt projections for *M. micrantha* under future climate scenarios reveal dynamic shifts in habitat suitability categories across the 2050s and 2070s, influenced by emission pathways (SSP1-2.6, SSP2-4.5, SSP3-7.0) (Figure 7; Table 1). On average, unsuitable areas experience a marginal contraction (−0.2%), while low-suitability areas decline notably (−13.6%), contrasted by expansions in moderate (14.0%), good (10.0%), and excellent (21.3%) categories. This pattern indicates an overall augmentation of suitability in the established range, with potential northward and inland advancements, particularly under higher emissions.

Under the low-emission SSP1-2.6 pathway, suitability maps show relative stability compared to current conditions. In the 2050s, excellent areas (4.3 × 10^4^ km^2^) remain anchored in southern strongholds, such as Hainan and the Leizhou Peninsula, with moderate expansions in the coastal lowlands of Guangdong and Guangxi. Low-suitability areas (14.8 × 10^4^ km^2^) recede slightly in the peripheral regions of Yunnan and Fujian. By the 2070s, excellent areas remain steady, but good areas decline to 8.8 × 10^4^ km^2^, suggesting minor contractions in transitional habitats, while moderate suitability persists in southeastern fringes.

The intermediate SSP2-4.5 scenario exhibits more pronounced changes. The 2050s projection features a modest increase in excellent habitat (4.1 × 10^4^ km^2^), with denser clusters of good and excellent suitability areas emerging in eastern Guangdong and southern Guangxi, alongside reduced low areas (13.9 × 10^4^ km^2^) in inland Yunnan. In the 2070s, excellent-suitability areas surge to 5.1 × 10^4^ km^2^, marking the largest gain among scenarios, with expansions penetrating northward into the borders of Jiangxi and Hunan. Good and moderate categories stabilize, but low suitability areas continue to shrink, indicating a shift toward higher-quality habitats in core tropical-subtropical zones.

The high-emission SSP3-7.0 drives the most substantial alterations. For the 2050s, moderate areas peak at 12.5 × 10^4^ km^2^, facilitating broader incursions into Fujian and Taiwan coastal belts, while excellent habitat (4.0 × 10^4^ km^2^) intensifies in southwestern Yunnan valleys. By the 2070s, the excellent category grows to 4.4 × 10^4^ km^2^, with notable northward extensions into the fringes of Guizhou and Sichuan, and a strengthened presence in the interior of Guangxi. Low-suitability areas drop sharply (11.7 × 10^4^ km^2^), reflecting conversion to more favorable conditions amid warming.

Overall, these forecasts highlight *M. micrantha*’s resilience and potential range expansion under climate change, with higher emissions accelerating notable suitability upgrades and some geographic shifts, posing increased invasion risks to northern subtropical ecosystems.

## 3. Discussion

### 3.1. Model Performance and Predictive Reliability

The MaxEnt model demonstrated superb predictive performance across all climate scenarios, with AUC values consistently exceeding 0.97, surpassing the threshold for excellent discrimination (AUC > 0.9). This high model accuracy aligns with findings from similar invasive plant studies in China. MaxEnt has proven particularly effective for predicting the potential distribution of exotic species with limited occurrence data [30,33,34]. Several factors contribute to this strong performance. First, the rigorous data quality control process, which filtered 2364 initial records to keep only 205 high-quality occurrence points by removing spatial duplicates within a 4.5 km radius, effectively reduced sampling bias while retaining a sufficient sample size for MaxEnt optimization. Previous studies have confirmed that MaxEnt achieves optimal accuracy with 75–100 occurrence records [36], suggesting our dataset size falls within the performance plateau range. Second, the systematic removal of multicollinear bioclimatic interpretability without sacrificing predictive power. Third, the ENMeval-based parameter optimization ensured selection of the most parsimonious model configuration based on minimum AICc [31,37], thereby balancing model fit and complexity.

However, interpreting model performance requires caution [38]. AUC values primarily indicate the model’s discrimination ability, that is, its capacity to distinguish presence sites from background locations. They do not directly evaluate calibration accuracy or spatial prediction precision [39]. Field validation surveys conducted in this study revealed general concordance between predicted high-suitability zones and actual invasion hotspots in Guangdong, Guangxi, and Hainan, lending empirical support to model projections. Nevertheless, discrepancies observed in some peripheral regions (e.g., sporadic occurrences in Sichuan and Chongqing) suggest that factors beyond the 19 bioclimatic variables considered here—such as soil properties, land-use patterns, propagule pressure, and biotic interactions—may influence local establishment success [13,15,40]. Future modeling efforts that incorporate these additional dimensions could further refine distributional predictions and enhance the applicability of management.

Although the present study deliberately focuses on climatic drivers to enable clear and interpretable projections across large spatial and temporal scales, non-climatic factors are known to strongly modulate actual realizations of climate-defined potential distributions, particularly at regional to local scales [13,41]. For example, intensive future land-use conversion (e.g., continued urbanization, road-network expansion, and agricultural intensification) in the predicted northward frontier zones (southern Jiangxi, Hunan, and Guizhou) is likely to create abundant disturbed habitats and increase propagule pressure, thereby facilitating establishment and accelerating invasion beyond what purely bioclimatic models suggest. Conversely, in topographically complex regions such as southwestern Yunnan and western Guangxi, rugged terrain and persistent forest cover may continue to act as dispersal barriers and refugia for native competitors, potentially constraining spread even in climatically suitable valleys. Soil properties (e.g., pH, fertility, and moisture retention) and biotic interactions (e.g., allelopathy from remaining native vegetation or herbivory) can further override climatic suitability at fine scales [42]. These interactions suggest that while our climate-only projections identify the maximum potential envelope and highlight zones deserving heightened surveillance, actual northward shifts may be either amplified by anthropogenic disturbance in lowland corridors or dampened in mountainous interiors. Integrating dynamic land-use/land-cover scenarios and soil data into future ensemble modeling will be crucial for translating broad-scale climatic risk into locally actionable management priorities.

### 3.2. Climatic Determinants of M. micrantha Distribution

The integrated analysis of percent contribution, permutation importance, and jackknife training gain unequivocally identifies annual mean temperature (Bio1) as the primary bioclimatic determinant of *M. micrantha* distribution in China, accounting for 84.3% of model contribution. This pronounced thermal dependency aligns with the species’ tropical origins in Central and South America [9,11], where it thrives in consistently warm environments with minimal seasonal variation. The sigmoid response curve, exhibiting a sharp inflection point near 20 °C and reaching optimal suitability above 25 °C, establishes a clear lower thermal threshold below which the species experiences severe physiological constraints on growth, reproduction, and overwintering survival [22]. This critical thermal requirement manifests geographically as a striking latitudinal constraint at approximately 27° N, corresponding to the bioclimatic transition from southern subtropical to central subtropical zones [43]. The current distribution pattern, concentrated almost entirely south of this threshold, reflects fundamental physiological limitations rather than dispersal barriers or propagule availability. Comparative studies on other tropical invasive species in China, including *Ageratina adenophora* and *Eupatorium odoratum* [44,45], have consistently identified temperature as the predominant limiting factor. However, *M. micrantha* exhibits greater temperature sensitivity than other tropical weeds, likely due to its evolutionary adaptation to lowland tropical forests, where freezing temperatures are absent and thermal fluctuations are minimal throughout the year.

Temperature seasonality (Bio4), ranking second in importance (5.4% contribution and 4.7% permutation importance), provides complementary insights, indicating that *M. micrantha* does not merely require warm conditions but demands thermal stability. The steep negative response curve, declining from optimal suitability at 150–400 standard deviation units to near-zero beyond 750 units, explains the species’ absence from interior regions of Yunnan and Guangxi, where annual mean temperatures may be adequate but strong monsoonal influences generate pronounced seasonal temperature amplitudes. In contrast, the coastal zones of Guangdong and Fujian, moderated by maritime influences, maintain relatively stable thermal regimes favorable for continuous growth and reproduction throughout the year. The unimodal response to maximum temperature of the warmest month (Bio5) peaks at 32–35 °C and drops sharply above 36 °C, revealing a clear upper thermal limit. Under the current climate, temperatures already exceed 36 °C for about 10 days per year in the hottest parts of the two excellent-suitability cores (Leizhou Peninsula and Pearl River Delta); yet these events remain short enough for the species to persist with only minor stress. By the 2070s under SSP3-7.0, the same locations are projected to experience more days per year above 36 °C, with some lowland grid cells reaching 40–42 °C during peak heatwaves. This prolonged exposure is expected to trigger heat-induced damage to photosystem II, accelerate leaf senescence, critically elevate respiratory costs, impair pollen viability, and amplify vapor-pressure-deficit-driven water stress [46,47,48], despite favorable annual mean conditions. Consequently, this upper thermal threshold, currently of marginal importance, will become a decisive limiting factor in parts of the present strongholds under the highest-emission pathway. The species’ otherwise strong heat tolerance and drought resistance are therefore insufficient to withstand such prolonged extreme heat [49].

While temperature variables dominate the bioclimatic niche, annual precipitation (Bio12) plays a critical secondary role, exhibiting disproportionately high permutation importance (5.9%) relative to its contribution (2.3%). This pattern, coupled with the variable’s substantial independent information gain in jackknife analysis, suggests that precipitation operates as a threshold filter rather than a gradual determinant [50]. The sigmoid response curve, reaching suitability thresholds (>0.50) at 1000–1200 mm and plateauing across 1500–4000 mm, indicates that while minimum moisture requirements must be met, the species demonstrates considerable tolerance to precipitation surplus above this baseline [51]. This flexible moisture requirement aligns with the species’ ecological strategy as a fast-growing, high-productivity invasive climber. Its rapid biomass accumulation and aggressive vegetative expansion necessitate sustained water availability, particularly during the growing season [10,24]. The concentration of high-suitability habitats in South China’s monsoon-dominated regions (annual precipitation 1200–2000 mm) reflects optimal moisture conditions. Additionally, precipitation of the driest month (Bio14), identified through jackknife analysis as producing substantial independent information gain, underscores the importance of inadequate moisture availability in the dry season. South China’s distinct wet-dry seasonal structure, with most precipitation concentrated in April–September [52], creates potential moisture stress during winter dry months. The species’ requirement for minimum dry-season precipitation can likely limit establishment in areas where prolonged drought coincides with suboptimal temperatures [49], compounding physiological stress from the dual water deficit and thermal constraints.

The relatively high permutation importance of mean diurnal temperature range (Bio2) (7.1%), despite low contribution (2.0%), reveals complex factor interaction effects, suggesting that while Bio2 provides limited independent predictive information, it becomes highly influential when other variables (particularly Bio1) are constrained. Ecologically, this effect suggests that near the 20 °C threshold, stable diurnal temperatures may favor establishment by mitigating thermal stress, whereas large diurnal ranges increase physiological costs through repeated heating-cooling cycles [53]. The complex interplay of these factors generates spatially heterogeneous suitability patterns across South China. On Hainan Island, where Bio1 exceeds 23 °C, Bio4 remains below 400 units, and Bio12 exceeds 1600 mm, all key variables converge within optimal ranges, producing consistently excellent suitability (>0.60) across lowland areas [54]. In contrast, Yunnan’s southwestern valleys exhibit suitable Bio1 and Bio12 but face constraints from moderate Bio4 values and topographic heterogeneity, resulting in fragmented suitable habitats that are restricted to thermally buffered valley floors [55]. Fujian’s coastal-inland gradient reflects the moderating effects of the maritime climate: coastal zones exhibit lower Bio4 and higher winter temperatures compared to interior regions, resulting in sharp transitions in suitability despite similar annual means. This regional variation highlights that *M. micrantha*’s distribution is shaped not by a single bioclimatic factor, but by synergistic interactions among temperature stability, thermal thresholds, and moisture availability. The resulting geographic patterns reflect the spatial product of these bioclimatic determinants across South China’s heterogeneous landscape.

### 3.3. Current Distribution Patterns and Invasion Dynamics

The current distribution of *M. micrantha* in China demonstrates a distinctly concentrated spatial structure, with Guangdong Province (including Hong Kong and Macau) serving as the primary invasion nucleus, followed by secondary strongholds in Hainan and Guangxi. This hierarchical pattern reflects the interplay of historical introduction passageways [19], propagule pressure, and landscape connectivity rather than climatic suitability alone [56]. Guangdong’s emergence as the invasion epicenter can be attributed to multiple synergistic factors beyond its favorable climate. First, the highly urbanized Pearl River Delta serves as China’s principal gateway for international trade and horticultural imports [57], serving as the likely primary introduction point for *M. micrantha* through ornamental plant markets, botanical gardens, and landscaping industries. This intense commercial hub with a long history facilitates frequent propagule influx and subsequent anthropogenic dispersal through nursery networks, contaminated soil transport, and landscape waste disposal [58]. Second, intensive land-use patterns characteristic of rapidly urbanizing regions generate abundant disturbance regimes—such as urbanization, road construction, forest fragmentation, riparian modification, and agricultural abandonment—that create invasion windows for this pioneer species, which is adapted to colonizing open and disturbed ruderal habitats [59]. Third, high population density and associated transportation infrastructure enable rapid human-mediated dispersal across the province [60], effectively bypassing natural dispersal limitations and accelerating range expansion. Near-complete spatial saturation occurs in Hong Kong and Guangdong. Occurrence records span coastal metropolises (Shenzhen, Guangzhou, Zhuhai), interior cities (Meizhou, Shaoguan, Qingyuan), and rural counties. This pattern indicates a successful transition from initial establishment to the landscape-scale expansion phase.

The invasion gradient observed across South China reveals stage-specific dynamics corresponding to distance from introduction centers and invasion history. In Guangxi, the pronounced occurrence concentrations in southern and coastal prefectures (Nanning, Beihai, Qinzhou, Fangchenggang, and Yulin) contrast sharply with sparse records in northern interior regions (Guilin and Baise), suggesting active expansion from Guangdong along coastal corridors with progressive attenuation inland and northward. This pattern likely reflects both climatic constraints at northern margins and time-lag effects [61], as the human-led invasion front has not yet penetrated interior landscapes despite their moderate suitability. Hainan Island exhibits nearly complete lowland occupation, with records extending from southern Sanya to northern Haikou and spanning both the eastern and western coasts, indicating a mature invasion status comparable to that of Guangdong. The conspicuous absence from central highlands (elevation >1000 m) provides field validation of the elevational limits predicted by MaxEnt, confirming that topographic barriers effectively constrain vertical spread [62]. In peripheral regions to the north of the species range, distinct invasion patterns emerge. Yunnan’s occurrences cluster tightly in southwestern border prefectures (Dehong, Xishuangbanna, Lincang, and Pu’er) [63], forming isolated population pockets separated by mountain ranges, which suggests dual invasion sources: historical introductions via trade routes and ongoing propagule influx from neighboring Myanmar and Laos populations. The failure to establish continuous populations across Yunnan despite many years since introduction indicates that topographic fragmentation and climatic heterogeneity impose significant dispersal barriers, constraining the species to localized linear refugia in thermally suitable valley floors [35,63]. Fujian’s records concentrate along the southeastern coast (Xiamen, Zhangzhou, and Fuzhou), forming a narrow linear coastal distribution that suggests recent northeastward expansion from Guangdong via coastal corridors, with the invasion front not yet penetrating interior regions.

Sporadic occurrences in climatically marginal provinces (Zhejiang, Jiangxi, Hunan, Chongqing, and Sichuan) form a distinct group that requires a different interpretation. These isolated records, typically comprising single individuals or small populations confined to botanical gardens, university campuses, or urban parks, likely represent independent anthropogenic introductions rather than natural range expansion from southern source populations [64]. Field validation conducted during this study confirmed that several such populations persist through microclimate subsidies (such as greenhouse cultivation, urban heat islands, and irrigation) or require periodic local in situ spontaneous re-invasion, lacking evidence of natural reproduction or recruitment. The conspicuous absence of establishment in surrounding natural or semi-natural habitats despite decades of presence supports the interpretation that these occurrences represent transient, human-maintained populations incapable of autonomous persistence under ambient climatic conditions. The MaxEnt model’s classification of these regions as unsuitable or low-suitability demonstrates accurate discrimination between viable invasion zones and climatically marginal areas where establishment requires anthropogenic subsidy.

By synthesizing distribution patterns, occurrence densities, population structures, and spatial continuity, we can classify the invasion dynamics of *M. micrantha* across China into three distinct stages. In Guangdong and Hainan, the species has reached a saturation stage, where it blankets the landscape with few gaps, maintains seamless connectivity between populations to enable genetic exchange and demographic support, occupies a wide array of habitats from urban and agricultural areas to natural ecosystems, and exerts clear, documented effects on native plant communities. Meanwhile, the less mature range of Guangxi and coastal Fujian is in an expansion stage, marked by solid regional footholds that are actively spreading outward, a dynamic distribution that is currently patchy but growing more interconnected over time. It is also marked by a focus on disturbed environments with early signs of encroachment into natural ones, and impacts that are noticeable yet not fully widespread. Finally, peripheral regions like Yunnan, interior Fujian, Taiwan, and isolated spots in other provinces remain in the establishment stage, featuring disconnected small populations confined to optimal microhabitats such as sheltered valleys or coastal lowlands, with little broader expansion even after decades, and virtually no significant ecological consequences.

### 3.4. Future Distribution Under Climate Change Scenarios

MaxEnt projections under future climate scenarios (2050s and 2070s) reveal a consistent pattern of quality-over-quantity expansion, wherein unsuitable areas contract marginally (−0.2%), low-suitability zones decline substantially (−13.6%), but moderate, good, and excellent categories increase notably (14.0%, 10.0%, and 21.3%, respectively). This pattern fundamentally differs from projections for some temperate-zone invasive species where climate warming enables dramatic poleward range expansions into previously unsuitable regions [65,66]. For thermophilic species like *M. micrantha* with stringent lower thermal thresholds (Bio1 > 20 °C), projected warming through the 21st century will remain insufficient to breach physiological limits across most of central and northern China, where annual mean temperatures will remain below critical establishment thresholds despite increases of 2–4 °C [67]. Instead, climate change primarily enhances suitability within the existing southern distribution envelope and nearby adjacent regions, effectively upgrading moderate-suitability transitional zones into good or excellent categories rather than expanding absolute geographic range. This in situ enhancement pattern suggests that invasion impacts will encounter spatial containment limiting their areal expansion, especially toward the north. Instead, the changes concentrate and amplify the effects in currently affected provinces rather than dispersing across broader landscapes. This largely in situ transformation, rather than notable area expansion into new sites, has important implications for management resource allocation and risk assessment frameworks.

The divergence among emission pathways becomes increasingly pronounced over time, particularly between projections for the 2050s and 2070s. Under the low-emission SSP1-2.6 scenario, excellent habitat increases modestly from 3.6 × 10^4^ km^2^ (current) to 4.3 × 10^4^ km^2^ in both 2050s and 2070s, representing relative stabilization compared to current conditions, with expansions concentrated in already invaded core regions (Pearl River Delta, and southern Guangxi coastal plains) rather than geographic range enlargements. Importantly, good-suitability areas slightly decline to 8.8 × 10^4^ km^2^ by the 2070s, suggesting that transitional habitats may undergo climate shifts [68]—such as greater precipitation variability or altered seasonality—not fully reflected in mean annual variables. This scenario implies that aggressive global emissions reductions could effectively constrain *M. micrantha* expansion, maintaining invasion intensity near current levels, and providing a viable pathway for limiting future ecological and economic damages [2,24,25].

The intermediate SSP2-4.5 scenario generates more substantial changes, particularly in the 2070s when excellent suitability surges to 5.1 × 10^4^ km^2^—the largest gain among all scenarios. Spatial analysis reveals two key trends: intensification in core tropical-subtropical zones where existing good-suitability areas in southern Guangdong and Guangxi upgrade to excellent, and northward penetration where moderate-to-good suitability expands into hitherto peripheral areas in southern Jiangxi and Hunan. The concurrent decline in low-suitability areas indicates consolidation into higher-quality categories rather than range contraction, suggesting that continued “business-as-usual” emissions trajectories could significantly enhance invasion risk across southeastern China and necessitate proactive management in currently invasion-free provinces [69].

The high-emission SSP3-7.0 scenario drives complex, non-linear responses that paradoxically do not always favor expansion. The 2050s projection shows moderate suitability peaking at 12.5 × 10^4^ km^2^ with expansions into the Fujian interior and the western lowlands of Taiwan. By the 2070s, excellent zones reach only 4.4 × 10^4^ km^2^—comparable to SSP1-2.6 and lower than SSP2-4.5 (5.1 × 10^4^ km^2^). This counter-intuitive contraction of top-tier habitat in the highest-emission scenario results from thermal overshooting, whereby the maximum temperature of the warmest month (Bio5) exceeds the species’ upper physiological tolerance. At such extremes, *M. micrantha* is likely to experience multiple stress pathways: (1) direct heat-induced damage to photo-system II and accelerated leaf senescence [70]; (2) sharply increased respiratory carbon loss that outpaces photosynthetic gains, leading to negative carbon balance during peak summer months [71]; (3) impaired pollen viability and seed set, as documented in many plants above 35 °C [47]; and (4) heightened water stress despite adequate annual precipitation, because extreme heat dramatically increases vapor pressure deficit and transpirational demand [48], particularly in the already seasonally dry winter–spring period of South China. These mechanisms collectively reduce growth, survival, and reproductive success in current core areas (especially the Leizhou Peninsula, Pearl River Delta, and lowland Guangxi), converting former excellent habitats into merely good or moderate ones by the late century under SSP3-7.0. This result highlights that, for strictly thermophilic invasive species originating from aseasonal tropical forests, both lower and upper thermal thresholds must be considered; unchecked warming may ultimately impose self-limiting feedback on their expansion in subtropical China [8,9]. This finding underscores the importance of considering both lower and upper thermal thresholds when projecting climate change impacts on thermophilic species [72].

Spatially explicit analysis reveals three primary distributional shifts that transcend simple latitudinal expansion patterns. First, the northern limit of good-to-excellent suitability shifts progressively from approximately 24° N (current) to 25–26° N (2050s) and potentially 26–27° N (2070s under SSP3-7.0), representing a 2–3° poleward encroachment that aligns with projected warming rates in subtropical China. Practically, this shift implies that provinces currently experiencing only sporadic invasions (Jiangxi, Hunan) may transition to climatically more suitable zones capable of supporting sustained establishment. This transformation necessitates the development of enhanced biosurveillance and early detection systems in these currently marginal regions [73]. However, the absolute northern boundary remains constrained by the 20 °C annual mean isotherm, which, even under high-emission scenarios, remains positioned around 27–28° N by the 2070s, preventing dramatic expansions into the Yangtze River basin or temperate provinces. Second, maritime provinces (Fujian, Guangdong coastal zones, and Taiwan) consistently exhibit suitability upgrades across all scenarios, reflecting synergistic effects of warming and the maintained moisture availability trait of maritime monsoonal climates [74]. Coastal ecosystems, already experiencing high invasion pressure, will face intensified impacts as climate change enhances the establishment success, growth rates, and competitive ability of *M. micrantha* populations, potentially accelerating the suppression and displacement of native coastal vegetation communities. Third, the interior regions of Yunnan and Guangxi display complex heterogeneous patterns, with some valley systems experiencing increases in suitability while others remain stable or decline. This spatial heterogeneity reflects topographic modulation of climate change signals [75]. The resulting landscape will likely feature increasingly fragmented suitability mosaics, complicating management planning and requiring spatially nuanced intervention strategies [7,9].

While the projections presented here provide valuable insights, several sources of uncertainty warrant cautious interpretation and suggest directions for further refinement. The reliance on a single General Circulation Model (BCC-CSM2-HR) limits the assessment of inter-model variability in regional climate predictions. Because different GCMs can produce substantially different estimates of temperature seasonality and monsoon-driven precipitation, incorporating multiple models would strengthen confidence in habitat suitability projections [76]. Although BCC-CSM2-HR performs well in simulating the East Asian monsoon and precipitation patterns over southern China [50,77,78], current best practice favors using multi-model ensembles from CMIP6 [79]. Such ensembles would better capture uncertainties in precipitation seasonality and dry-season moisture—variables critical to *M. micrantha*—and may reveal potential high-risk areas or divergent future scenarios overlooked by a single-model approach. Second, the assumption of static species–climate relationships may oversimplify future responses [80], as *M. micrantha* may express evolutionary adaptation or phenotypic plasticity under novel environmental conditions over multi-decadal timescales. Third, the exclusion of potential CO_2_ fertilization effects could lead to a conservative assessment of future performance for this C_3_ species under elevated CO_2_ concentrations expected in SSP2-4.5 and SSP3-7.0 scenarios [81]. Finally, the omission of extreme climatic events—such as heatwaves, droughts, floods, and typhoons—from WorldClim’s 30-year mean climatic regimes may limit the model’s capacity to capture episodic disturbances that may strongly influence invasion dynamics [82]. Future studies integrating ensemble GCMs, mechanistic physiological models, evolutionary dynamics, and extreme-event simulations would enhance predictive robustness and support more adaptive management planning.

### 3.5. Implications for Invasion Management

The integration of current distribution, habitat suitability, and future projections provides a foundation for a spatially explicit, risk-based management framework for *M. micrantha*. Based on invasion stages and climatic suitability, management priorities can be regionally differentiated. In severely invaded areas such as Guangdong and Hainan, where the species has achieved landscape-level dominance, containment and mitigation should be emphasized. Actions should focus on preventing further spread to neighboring provinces, minimizing ecological impacts in biodiversity-rich areas, and applying integrated control measures that combine mechanical removal, selective herbicide use, and cautiously assessed biocontrol options [18,20,21]. Long-term monitoring of community-level impacts is crucial for evaluating the effectiveness of management and informing adaptive strategies [83].

In prospective expansion-phase regions such as Guangxi, Fujian, and the coastal area of southeast China, where populations remain localized but are spreading, management should prioritize early suppression. Aggressive eradication of newly detected populations, continuous monitoring of high-risk habitats (e.g., riparian zones and disturbed sites) [59], and rapid-response mechanisms are critical to preventing the formation of landscape connectivity [84]. Public education and regulatory control of plant trade can reduce human-mediated dispersal, representing a cost-effective intervention before invasions become irreversible [85].

In early detection regions, including Sichuan, interior Yunnan, and peripheral provinces, surveillance networks should target potential introduction pathways such as transport corridors and horticultural centers [86]. Rapid-response systems with pre-positioned resources and restoration of native vegetation in disturbed areas can enhance ecosystem resistance [84]. In currently invasion-free areas, such as southern Jiangxi, Hunan, and Guizhou, preventive and preemptive measures—including baseline biodiversity assessments, training for land managers, and biosecurity inspections—should be established to reduce future invasion risk [9].

Despite significant progress in modeling invasion risk [73,87], several knowledge gaps still constrain the refinement of management strategies. Long-term demographic research is necessary to elucidate the impact of climatic factors on population growth and survival [83]. Studies on dispersal ecology and biotic interactions would improve predictions of spread potential and control effectiveness [88]. Experimental analyses of responses to climate extremes—such as droughts and cold spells—could refine understanding of physiological limits in marginal habitats [89]. Investigations into adaptive differentiation among populations would indicate whether *M. micrantha* can evolve tolerance to novel environmental conditions. Furthermore, integrating socioeconomic research—assessing costs, public perceptions, and the impacts on ecosystem services—would enhance both the efficiency and public acceptance of management programs.

Climate change will substantially reshape invasion risk landscapes, though not necessarily through poleward range shifts. For thermophilic species like *M. micrantha*, warming primarily intensifies invasion pressure within existing ranges rather than enabling large-scale expansion. Consequently, resource allocation should prioritize containment and suppression within current and adjacent invasion zones, where management remains most effective. The contrast among emission scenarios also highlights that global climate mitigation serves as an indirect but powerful invasion management strategy. Under low-emission scenarios, projected expansion remains modest, whereas high-emission pathways greatly elevate risk and management costs. As climate change accelerates and invasion pressures intensify, such integrated, climate-adaptive frameworks will be essential for anticipatory and cost-effective management of biological invasions.

## 4. Materials and Methods

### 4.1. Species Occurrence Data

Data on *M. micrantha* occurrence were compiled from multiple sources to ensure comprehensive coverage and reliability. These included the National Specimen Information Infrastructure (http://www.nsii.org.cn/2017/home.php (accessed on 12 May 2024); 120 records), the Plant Photo Bank of China (http://ppbc.iplant.cn/ (accessed on 17 June 2024); 1942 records), field surveys conducted in potential habitats across South China (117 records), online news reports from credible outlets documenting sightings (61 records), and literature from the China National Knowledge Infrastructure (CNKI; 124 records). To incorporate global perspectives and enhance data robustness, additional records were sourced from the Global Biodiversity Information Facility (GBIF; https://www.gbif.org/(accessed on 21 March 2024); 150 records) and the Chinese Virtual Herbarium (CVH; https://www.cvh.ac.cn/(accessed on 11 June 2024); 85 records). A total of 2649 records were assembled.

To ensure data quality, minimize spatial autocorrelation, and prevent overfitting, a rigorous cleaning process was applied. Duplicate records, those lacking precise geographic coordinates (latitude and longitude), and closely spaced points (within a 4.5 km radius, corresponding to the environmental data resolution) were removed using spatial filtering in QGIS 3.42 software [90]. Erroneous or outlier points were identified and excluded based on expert review and cross-verification with known ecological ranges. The rigorous screening resulted in 205 high-quality, spatially independent occurrence records suitable for modeling, as visualized in a distribution map for validation.

### 4.2. Bioclimatic Variables

Current climate data (1970–2000) were obtained from WorldClim version 2.1 (www.worldclim.org, (accessed on 23 April 2021)) at a spatial resolution of 2.5′ [82], comprising 19 bioclimatic variables (Bio1–Bio19) that capture temperature and precipitation patterns critical for plant distribution. Future climate projections for the 2050s (2041–2060) and 2070s (2061–2080) were derived from the IPCC Sixth Assessment Report (AR6) using the BCC-CSM2-MR global climate model under three Shared Socioeconomic Pathways (SSPs): SSP1-2.6 (low emissions, sustainable development), SSP2-4.5 (intermediate emissions, middle-of-the-road), and SSP3-7.0 (high emissions, fossil-fueled development) [78]. These scenarios represent a range of greenhouse gas emission trajectories to assess uncertainty in future distributions.

To mitigate multicollinearity and improve model transferability, bioclimatic variables were screened using a multi-step approach. First, Pearson correlation coefficients were calculated using PAST (version 4.12b) [91], retaining variables with |r| < 0.8. For highly correlated pairs (|r| ≥ 0.8), variables with greater ecological relevance to *M. micrantha* (e.g., based on literature on invasive vines) were prioritized [28]. The jackknife test in MaxEnt was employed to evaluate variable importance, percent contribution, and permutation importance [30]. Response curves for key variables (e.g., annual mean temperature, annual precipitation) were generated to interpret their ecological influence on *M. micrantha* suitability, revealing thresholds and optima [92]. Ultimately, eight variables were selected: Bio1 (annual mean temperature), Bio2 (mean diurnal range), Bio4 (temperature seasonality), Bio5 (max temperature of warmest month), Bio8 (mean temperature of wettest quarter), Bio12 (annual precipitation), Bio14 (precipitation of driest month), and Bio15 (precipitation seasonality).

### 4.3. Model Optimization

The MaxEnt model (version 3.4.4) was optimized to strike a balance between complexity and predictive accuracy, thereby reducing overfitting while capturing ecological patterns. Using the ENMeval package (version 2.0.3) in R [37], regularization multipliers (RM) were tested across eight values (0.5 to 4.0, in 0.5 increments). Six feature class combinations were evaluated: L (linear), LQ (linear + quadratic), H (hinge), LQH (linear + quadratic + hinge), LQHP (linear + quadratic + hinge + product), and LQHPT (linear + quadratic + hinge + product + threshold). This yielded 48 parameter combinations. The Checkerboard2 spatial partitioning method was applied to divide occurrence data into four bins, enhancing regularization assessment across geographic space. Model performance was evaluated using the delta Akaike Information Criterion corrected for small sample sizes (delta.AICc), with the optimal parameters selected where delta.AICc = 0 (Wu et al. 2025), alongside checks for low omission rates (10% training omission rate) to ensure generalizability [31].

### 4.4. MaxEnt Modeling and Evaluation

The filtered occurrence data for *M. micrantha* and selected bioclimatic variables (under current and future scenarios) were input into the optimized MaxEnt model to predict potential distributions across China. The dataset was randomly partitioned into 60% for training and 40% for testing [30], with a maximum of 5000 background points, 5000 iterations for convergence, and 10-fold cross-validation to assess robustness. Model outputs in logistic format (representing habitat suitability from 0 to 1) were exported as ASCII grids and converted to raster layers in QGIS. Habitat suitability was classified using the natural breaks (Jenks) method for ecologically meaningful thresholds [29]: unsuitable (0.00–0.15), low (0.15–0.30), moderate (0.30–0.45), good (0.45–0.60), and excellent (>0.60). Suitable habitat areas (in km^2^) were quantified for each period and SSP scenario using ‘Reclassify by table’ in QGIS. Model performance was evaluated with the area under the receiver operating characteristic curve (AUC). AUC values were interpreted as: 0.9–1.0 = excellent; 0.8–0.9 = good; 0.7–0.8 = fair; 0.6–0.7 = poor; <0.6 = failed [28,31].

## 5. Conclusions

This study demonstrates that climate change will significantly alter the invasion dynamics of *M. micrantha* in China, with future warming expected to enhance habitat suitability within already invaded southern regions rather than drive extensive poleward range expansion. Enhanced climatic favorability in these core areas is likely to foster denser and more persistent populations, furnishing more bridgeheads to facilitate dispersal, and exacerbating ecological degradation and agricultural losses under high-emission scenarios. The species’ pronounced sensitivity to temperature variability and dependence on warm, humid conditions underscore the necessity of integrating climate projections into management planning. Scenario-dependent patterns further reveal that global emission trajectories will play a decisive role in shaping future invasion risks: low-emission pathways may constrain range expansion, while high-emission scenarios substantially elevate invasion pressure and management complexity.

Building upon these insights, this work advocates a climate-adaptive and spatially differentiated management framework. Priority actions include reinforcing containment and suppression efforts in established hotspots through integrated mechanical, chemical, and biological control measures; enhancing early detection and rapid-response capacity in frontier regions via remote sensing and real-time monitoring; and promoting large-scale ecological restoration to strengthen natural resistance against reinvasion. Achieving long-term success requires synergistic policy frameworks that integrate predictive modeling with adaptive governance, ensuring that management strategies remain flexible in response to shifting climatic and ecological conditions. Continued interdisciplinary research on *M. micrantha*’s evolutionary potential, dispersal ecology, and responses to multiple stressors will further refine predictive accuracy and inform evidence-based, resilient strategies for invasive species management in a changing world.

## Figures and Tables

**Table 1:** Predicted suitable habitat areas (×10^4^ km^2^) for *Mikania micrantha* under current and future climate scenarios are classified into five suitability categories generated by the MaxEnt model.

Climate Scenario	Unsuitable	Low	Moderate	Good	Excellent
Current	924.5	15.9	10.1	8.3	3.6
SSP1-2.6 (2050s)	921.1	14.8	11.9	9.6	4.3
SSP1-2.6 (2070s)	922.5	14.4	11.7	8.8	4.3
SSP2-4.5 (2050s)	923.4	13.9	11.5	8.9	4.1
SSP2-4.5 (2070s)	924.0	13.8	9.8	9.0	5.1
SSP3-7.0 (2050s)	922.5	13.8	12.5	9.0	4.0
SSP3-7.0 (2070s)	924.4	11.7	11.7	9.5	4.4
Average ^a^	923.0	13.7	11.5	9.1	4.4
Change ^b^ (%)	−0.2	−13.6	14.0	10.0	21.3

^a^ The average value of suitable habitat areas projected under future climate change scenarios. ^b^ Comparison of suitable habitat areas under the current climate scenario and the average values projected under future climate change scenarios.

## Data Availability

Data in this study are available at https://doi.org/10.57760/sciencedb.19837, accessed on 20 January 2025.

## Appendix A

**Table A1:** Nineteen bioclimatic variables initially included to predict the distribution patterns of *Mikania micrantha*. The eight highlighted variables were selected by a multicollinearity test for MaxEnt modeling.

Bioclimatic Variable	Unit	Bioclimatic Variable	Unit
**Bio1: Annual mean temperature**	°C	Bio11: Mean temperature of the coldest quarter	°C
**Bio2: Mean diurnal range**	°C	**Bio12: Annual precipitation**	mm
Bio3: Isothermality	Index	Bio13: Precipitation of the wettest month	mm
**Bio4: Temperature seasonality**	Index	**Bio14: Precipitation of the driest month**	mm
**Bio5: Max temperature of the warmest month**	°C	**Bio15: Precipitation seasonality**	Index
Bio6: Min temperature of the coldest month	°C	Bio16: Precipitation of the wettest quarter	mm
Bio7: Temperature annual range	°C	Bio17: Precipitation of the driest quarter	mm
**Bio8: Mean temperature of the wettest quarter**	°C	Bio18: Precipitation of the warmest quarter	mm
Bio9: Mean temperature of the driest quarter	°C	Bio19: Precipitation of the coldest quarter	mm
Bio10: Mean temperature of the warmest quarter	°C		
